# The Development of a One-Step RT-qPCR for the Detection and Quantification of Viable Forms of *Trypanosoma cruzi* in Açai Samples from Areas at Risk of Chagas Disease through Oral Transmission

**DOI:** 10.3390/ijms25105531

**Published:** 2024-05-18

**Authors:** Amanda Faier-Pereira, Paula Finamore-Araujo, Carlos Ramon do Nascimento Brito, Eldrinei Gomes Peres, Klenicy Kazumy de Lima Yamaguchi, Daniele Pereira de Castro, Otacilio C. Moreira

**Affiliations:** 1 Laboratory of Molecular Virology and Parasitology, Oswaldo Cruz Institute, Fiocruz, Rio de Janeiro 21040-360, Brazil; 2 Departament of Clinical e Toxicological Analysis, Federal University of Rio Grande do Norte, Natal 59078-970, Brazil; 3 Departament of Chemistry, Federal University of Amazonas, Manaus 69067-005, Brazil; 4 Institute of Health and Biotecnology, Federal University of Amazonas, Coari 69460-000, Brazil; 5 Laboratory of Biochemistry and Physiology of Insects, Oswaldo Cruz Institute, Fiocruz, Rio de Janeiro 21040-360, Brazil

**Keywords:** Chagas disease, oral transmission, açaí, *T. cruzi*, one-step RT-qPCR, viability

## Abstract

Currently, approximately 70% of new cases of Chagas disease (CD) in Brazil are attributed to oral transmission, particularly through foods such as açaí, bacaba, and sugarcane juice, primarily in the northern and northeastern regions of the country. This underscores the imperative need to control the spread of the disease. The methods utilized to conduct quality control for food associated with outbreaks and to assess the potential for the oral transmission of CD through consuming açaí primarily rely on isolating the parasite or inoculating food into experimental animals, restricting the analyses to major research centers. While there are existing studies in the literature on the detection and quantification of *T. cruzi* DNA in açaí, the evaluation of parasites’ viability using molecular methods in this type of sample and differentiating between live and dead parasites in açaí pulp remain challenging. Consequently, we developed a molecular methodology based on RT-qPCR for detecting and quantifying viable *T. cruzi* in açaí pulp samples. This protocol enables the stabilization and preservation of nucleic acids in açaí, along with incorporating an exogenous internal amplification control. The standardization of the RNA extraction method involved a simple and reproducible approach, coupled with a one-step RT-qPCR assay. The assay underwent validation with various *T. cruzi* DTUs and demonstrated sensitivity in detecting up to 0.1 viable parasite equivalents/mL in açaí samples. Furthermore, we investigated the effectiveness of a bleaching method in eliminating viable parasites in açaí samples contaminated with *T. cruzi* by comparing the detection of DNA versus RNA. Finally, we validated this methodology using açaí pulp samples positive for *T. cruzi* DNA, which were collected in a municipality with a history of oral CD outbreaks (Coari-AM). This validation involved comparing the detection and quantification of total versus viable *T. cruzi*. Collectively, our findings demonstrate the feasibility of this methodology in detecting viable forms of *T. cruzi* in açaí pulp samples, emerging as a crucial tool for monitoring oral outbreaks of Chagas disease resulting from açaí consumption.

## 1. Introduction

Chagas disease (CD) is one of the main public health problems in Latin America [1,2]. Countries such as Bolivia, Argentina, Paraguay, Ecuador, El Salvador, and Guatemala have the highest numbers of infected individuals of the 21 countries that are considered endemic [3,4]. Currently, it is estimated that 6–7 million people worldwide are infected, and about 75 million are exposed to the risk of infection [5]. The parasite can be transmitted to humans by mechanisms other than classic vectorial transmission, such as blood transfusion, vertically from mother to child, through accidents in laboratories, or through the ingestion of food or liquids contaminated with viable *T. cruzi* [3]. Oral transmission can occur through the consumption of food contaminated with triatomines and/or their feces, including juices and pulps such as those from sugar cane, bacaba, and açaí, or even through the consumption of raw meat from mammals, which may be infected wild hosts [6,7]. *T. cruzi* infection in fresh foods is typically accidental and can occur during harvesting, storage, transport, or preparation [8,9].

In Brazil, the oral transmission of the parasite is responsible for the increase in morbidity and mortality caused by Chagas disease. Between 2000 and 2011, 1252 cases of acute Chagas disease were reported in the country, of which 70% were attributed to oral transmission [10,11,12,13], and according to more recent data from the Epidemiological Bulletin of Chagas disease (2021), oral transmission cases reached 75.34% in 2020 in Brazil, predominantly in the northern region of the country.

Açaí pulp has excellent nutritional properties and is popular in Brazil and abroad [8]. Studies point out that, when collecting açaí, the parasite can be accidentally collected and make contact with uncovered pans used for pulp production, and pulp is marketed and consumed every day in natural or frozen forms [14]. Furthermore, the insect is attracted by the reflection of light on the fruit [15] and by the odors produced during fermentation, which is accelerated by heat and humidity, favoring deterioration and natural contamination [16]. Therefore, the lack of hygiene during the harvesting and handling stage of the fruit is one of the main targets of *T. cruzi* contamination, leading açaí to be the food associated with the highest number of cases of oral outbreaks of CD in the Brazilian Amazon region in recent years [12,17]. The use of PCR for the detection of *T. cruzi* DNA from açai has already been described in the literature, specifically in studies showing that the molecular detection technique has greater sensitivity compared to other detection methods [9,18,19,20,21,22,23]. However, a recurrent issue regarding the use of DNA for pathogen detection is the correlation with its viability [24,25]. Evaluating the presence of DNA sequences alone does not prove the pathogen’s viability or infectious capacity, since a DNA molecule is stable and easily amplified by the PCR method [26]. Therefore, because the robustness of DNA and amplification signals are not necessarily related to the detection of viable infectious parasites, there has recently been an interest in the use of RNA as the molecular target for viability assessments. Both mRNA and rRNA have a shorter half-life compared to DNA, making them potentially superior indicators of viable organisms [27,28,29]. Thus, assays utilizing mRNA amplification, such as RT-PCR and RT-qPCR techniques, are likely to indicate the presence of live parasites, serving as potential indicators of parasite susceptibility. The RNA molecule, as it is more labile, has been widely used as a viability marker in other models of host–pathogen interaction or in tumor cells [30,31]. However, there are still few molecular diagnostic studies that demonstrate the performance of the RNA molecule as a viability marker, especially for the evaluation of *T. cruzi* in different biological matrices such as food samples involved in oral CD outbreaks [31,32]. Previously, we developed and standardized an RT-qPCR assay to assess the viability of *T. cruzi* in the *Rhodnius prolixus* triatomine vector, which was artificially fed with blood contaminated with *T. cruzi* [31]. This study enabled the comparison between the detection and quantification of *T. cruzi* DNA and mRNA, evaluating parasite RNA as a viability marker in the parasite–vector interaction.

Initially, we confirmed that RT-qPCR was an effective tool in detecting viable forms of *T. cruzi* by conducting an experiment where the parasite was lysed by heating and monitored for up to 3 days post-lysis. In this experiment, we observed that mRNA was rapidly degraded and not detected shortly after lysis, while DNA was detected and remained stable for up to 3 days post-lysis. To confirm that the lysed parasites (which did not show detectable RNA) were not viable, they were inoculated into a culture medium (LIT) enriched with fetal bovine serum. This culture was monitored for up to 10 days. In parallel, a control was performed with parasites that did not undergo thermal lysis. It was observed that the lysed parasites did not grow in the culture medium, indicating complete inactivation. In contrast, parasites from the control group grew in the culture medium, reaching the stationary growth phase after 7 days.

By comparing the detection of parasite mRNA with DNA in the intestine of *R. prolixus* artificially fed with blood contaminated with *T. cruzi*, we observed a statistically significant decrease in the quantity of *T. cruzi* mRNA 15 days after feeding, which occurred more rapidly than the decrease in the parasite load observed by DNA detection. This was likely the result of the insect’s immune response, which led to the lysis of a large number of parasites in the first two weeks after feeding on blood contaminated with *T. cruzi*. Thus, the number of detected viable parasites through *T. cruzi* mRNA was lower than the number of total, live, and dead parasites detected through *T. cruzi* DNA, confirming that mRNA, detected and quantified by RT-qPCR, is a potential viability marker for *T. cruzi*.

Thus, in this work, we developed and validated a methodology based on silica column RNA extraction using a synthetic exogenous internal positive control RNA capable of validating the quality of the sample and the absence of PCR inhibition, discarding false negative results. Additionally, we validated a one-step RT-qPCR assay to detect and quantify viable *T. cruzi* in açaí pulp samples in a fast, sensitive, and reproducible way in order to contribute to the surveillance and follow-up of CD oral outbreaks caused by the ingestion of *T. cruzi*-contaminated açaí.

## 2. Results

### 2.1. The Establishment of the RNA Exogenous Internal Positive Control

By aiming to control the quality of the RNA extracted from açaí pulp that was previously stabilized in a guanidine–EDTA solution (GEA), regarding the presence of possible PCR inhibitors and variations in the extraction yield, the use of an exogenous internal control of RNA (RNA-IAC) was established. In addition, through RNA-IAC amplification, it is possible to differentiate true negative from false negative samples. It is an exogenous synthetic RNA corresponding to the transcript of the firefly (Coleoptera) luciferase gene, which has no homology with the transcripts of açaí nor *T. cruzi*. Five microliters of RNA-IAC (10^6^ copies/uL) was added to the GEA samples prior to RNA extraction, as described in the Materials and Methods Section. The quality of RNA was evaluated through the amplification of RNA-IAC in a one-step RT-qPCR assay, which was performed in parallel to the *T. cruzi* RNA assay.

**Figure 1:**
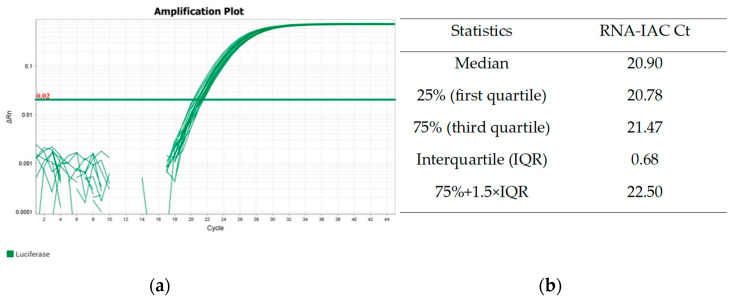
Quality control of RNA from GEA samples through amplification of RNA-IAC. GEA samples were spiked with RNA-IAC prior to extraction. In (**a**), amplification plots from RT-qPCR targeting RNA-IAC are shown. (**b**) shows statistics of RNA-IAC Ct values. To obtain the Ct values, threshold was set at 0.02.

In Figure 1a, it is possible to observe the amplification curves of nine RNA samples extracted from GEA in the same extraction batch. The Ct values for the RNA-IAC target showed little variation, increasing from 19.45 to 20.74. The median of the Ct values was 20.90 with interquartile ranges (first and third quartiles) of 20.78 and 21.47, respectively (Figure 1b). The calculation of the cut-off for identifying outliers was performed using Tukey’s range test (75% + 1.5 × IQR), reaching the value of 22.50 for this extraction batch. Thus, as no Ct value greater than this was observed, all samples were approved for subsequent analyses.

### 2.2. Analytical Sensitivity for Viable T. cruzi Detection

In order to evaluate the analytical sensitivity for viable *T. cruzi* detection, GEA samples were spiked with viable *T. cruzi* (TcI-Dm28c clone), reaching from 5 to 0.1 parasite equivalents/mL, before RNA extraction. *T. cruzi* and RNA-IAC amplifications were monitored via Ct values in one-step RT-qPCR assays. As shown in Table 1, it was possible to observe the amplification of *T. cruzi* in all concentrations tested, with the Ct values ranging from 28.45 ± 0.38 to 34.27 ± 0.01, up to the detection of 0.1 *T. cruzi* equivalents/mL. Furthermore, the Ct values for RNA-IAC only varied from 22.52 ± 0.06 to 23.54 ± 0.28 regardless of the *T. cruzi* concentration in the samples, and no outlier was observed.

### 2.3. The Reportable Range of Viable T. cruzi Detection in the One-Step RT-qPCR Assay (Linearity Assay)

**Figure 2:**
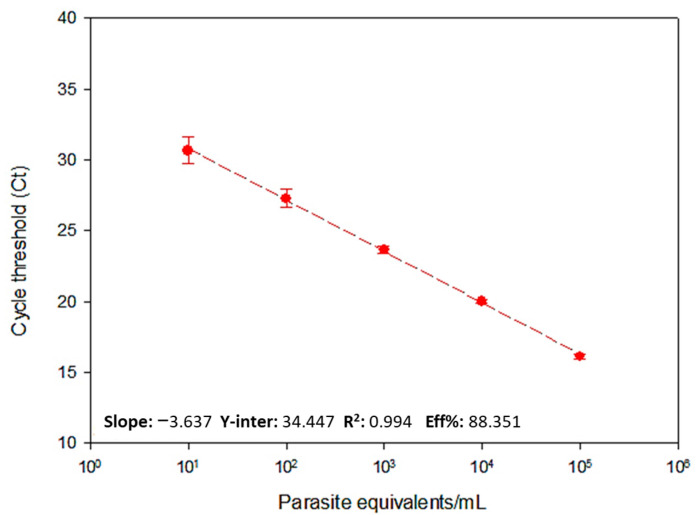
The reportable range for quantification of *T. cruzi* in açaí samples by one-step RT-qPCR. A ten-fold serial dilution of RNA extracted from GEA spiked with *T. cruzi* was used to generate the standard curve for the *T. cruzi* target, ranging from 10^5^ to 10 Par. Eq./mL. The standard curve parameters are shown at the bottom of the graph.

To assess the reportable range (dynamic extension) of *T. cruzi* RNA amplification by one-step RT-qPCR, RNA from GEA samples spiked with *T. cruzi* was serially diluted into RNA from negative GEA. Thus, it was possible to observe a linearity of 10^5^ to 10 Par. Eq./mL, with an RT-qPCR efficiency of 88.4% for the amplification of *T. cruzi* RNA and a coefficient of determination of 0.99, as shown in Figure 2. This result made it possible to accurately quantify the viable *T. cruzi* load (RNA) in the GEA samples containing different parasite loads.

### 2.4. Detection of Viable T. cruzi from Different DTUs by One-Step RT-qPCR (Inclusivity Assay)

The ability of the one-step RT-qPCR assay to amplify *T. cruzi* RNA from different DTUs, especially those most related to oral outbreaks due to açaí consumption in northern Brazil (TcI, TcIII, and TcIV), was investigated. Table 2 shows that the one-step RT-qPCR was able to detect the three *T. cruzi* DTUs tested, with concentrations ranging from 10^4^ to 0.1 Par. Eq./mL, except for TcIII, which was only possible to detect *T. cruzi* at a concentration up to 10 Par. Eq./mL. However, the exo-IAC was amplified for all concentrations tested, with Ct values ranging from 24.74 ± 0.28 to 26.85 ± 0.05 for TcIII, validating the negative results. Together, all the DTUs tested showed mean Ct values for *T. cruzi* ranging from 20.83 ± 0.98 (for TcI) to 40.08 ± 4.31 (for TcIV).

### 2.5. Precision on the Detection of T. cruzi RNA by One-Step RT-qPCR

The precision (repeatability) of the one-step RT-qPCR assay on the detection of *T. cruzi* RNA by the same operator was evaluated. For this, based on the analytical sensitivity results, three *T. cruzi* concentrations were selected: 2, 1, and 0.5 Par. Eq./mL. Forty technical replicates were tested at each concentration, and the results were compared (Table 3). The amplification of all replicates (100%) was observed at concentrations of 2 and 1 Eq. Par/mL, and 97.50% was observed at the lowest concentration (0.5 Eq. Par/mL) with mean Ct (±Standard Deviation) values of 30.91 ± 0.54, 32.07 ± 0.48, and 32.47 ± 0.65, respectively, and coefficients of variation of 1.74%, 1.49%, and 2.01%, respectively. All the coefficients of variation were below 5.0%, which means there was high precision in the measurements of *T. cruzi* RNA, even in low concentrations. 

### 2.6. The Validation of the One-Step RT-qPCR Assay with Açai Samples from the Field

Previously [20], we evaluated samples of açaí pulp collected at points of sale in the municipality of Coari (State of Amazonas, northern region of Brazil), which has a history of oral outbreaks of Chagas disease. From the nine GEA samples that were positive for *T. cruzi* DNA, and which were stored at −80 °C, the RNA was extracted, and the presence of viable *T. cruzi* (at the time of collection and preservation in the guanidine–EDTA) was investigated by one-step RT-qPCR assays according to our methodology.

**Figure 3:**
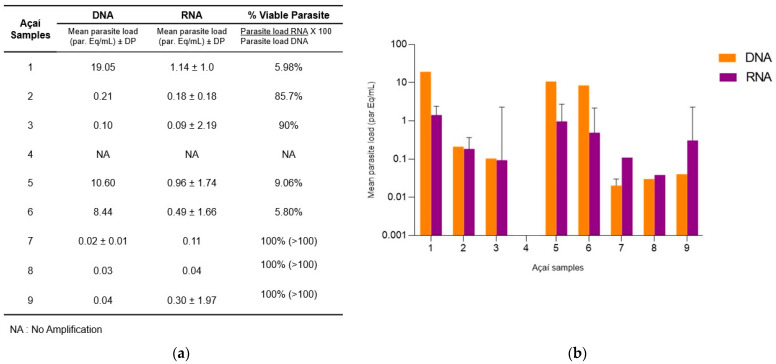
RT-qPCR assays for the detection and quantification of viable parasites in açai pulp samples. Nine açaí pulp samples that were positive for *T. cruzi* DNA were obtained from different points of sale in the municipality of Coari (Amazonas, Brazil). *T. cruzi* DNA and RNA were detected and quantified according to our methodology in the same GEA sample (**a**). The table shows the parasite load results, reported as the mean parasite load (parasite equivalents/mL) ± SD and the percentage of viable parasites. (**b**) A comparative bar chart of parasite loads ± SD estimated from DNA and RNA. Samples with an RNA parasite load higher than the DNA parasite load were considered 100% viable parasites.

Except for sample 4, in all other samples, it was possible to detect *T. cruzi* RNA (Figure 3). The viable parasite load (RNA) ranged from 0.04 to 1.14 Eq. Par./mL. Based on the estimated parasite load ratio for DNA and RNA, it was possible to calculate the percentage of viable parasites in the sample [(Parasite Load RNA/Parasite Load DNA) × 100] (Figure 3a). In Figure 3b, it is possible to observe the differences in parasite load between the measurements performed with DNA and RNA. The percentage of viable parasites ranged from 0 to 100%, with a median of 9.1 [5.8–90.3]%, indicating that some samples had viable parasites at the moment of harvesting.

### 2.7. Quantification of T. cruzi DNA and RNA in Bleached Açai Samples

**Figure 4:**
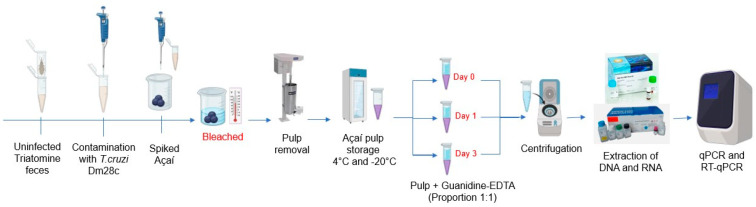
The experimental design of the bleaching step. The açaí fruits were artificially contaminated with triatomine feces enriched with *T. cruzi*, followed by bleaching of the fruits before preparing the pulp. Then, the açaí pulp was stored at 4 °C and −20 °C (for up to three days), the DNA and RNA were extracted, and finally, the qPCR and RT-qPCR were performed.

Aiming to evaluate whether our methodology would be able to differentiate viable from non-viable parasites by the quantification of RNA and DNA using real-time PCR assays, we simulated the açaí bleaching step, which is recommended by the Brazilian national health surveillance agency (ANVISA) and commonly used in açaí pulp sale points in the northern region of Brazil. For this, we artificially contaminated the açaí fruits with triatomine feces spiked with *T. cruzi* and performed the bleaching of the açaí fruits before pulp preparation (Figure 4). After bleaching, the açaí pulps were stored at 4 °C or −20 °C for up to three days before DNA and RNA extraction according to our methodology.

**Figure 5:**
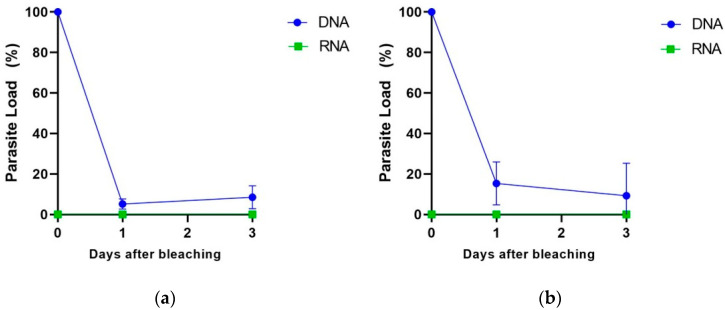
Comparison of parasite load quantified using *T. cruzi* DNA and RNA in açai pulp samples after bleaching. Açai pulp samples were stored at 4 °C (**a**) or −20 °C (**b**) for up to 3 days after bleaching. DNA and RNA were extracted from açaí pulp, and qPCR and one-step RT-qPCR were performed for detection and quantification of total or viable parasite load, respectively. Total parasite load (DNA) is represented by blue circles, and viable parasite load (RNA) is represented by green squares, in percentage of control (time zero).

We observed that, even after bleaching the contaminated açaí fruits and preparing the pulp, it was no longer possible to detect viable parasites by RNA quantification, regardless of the pulp storage temperature (Figure 5a,b). On the contrary, DNA was detected at all evaluated storage times and temperatures. By considering the total parasite load of DNA right after bleaching (zero time) as 100%, we observed a decrease in the parasite load to 10% after 1 and 3 days of storage at 4 °C (Figure 5a). When stored at −20 °C, a less pronounced decrease in the amount of *T. cruzi* DNA was observed: there were 20% and 10% decreases after 1 and 3 days of storage, respectively (Figure 5b). All of the Ct values for this experiment, for DNA and RNA detection and including the internal controls, are reported in Table S1. No false negative result was observed for RNA detection. These results suggest that *T. cruzi* RNA can act as a marker of parasite viability in açaí pulp samples in contrast to DNA, which remains detectable even after açai pulp bleaching.

## 3. Discussion

Currently, the detection of *T. cruzi* in different samples is carried out by traditional parasitological methods, such as cultivation followed by microscopic observation. However, these methods are laborious, time-consuming, and more subject to contamination [33]. Some studies were effective in identifying *T. cruzi* in food samples [9,18,20,21,22,23,34]. Recently, our group developed an easily, reproducible, fast, and sensitive multiplex qPCR method that is capable of identifying the parasite’s DNA in açaí pulp samples [19]. However, the evaluation of the presence of *T. cruzi* DNA in the açaí sample does not indicate the parasite’s viability or infectious capacity since the parasite’s DNA can be found in the sample even after its sterilization. In contrast, the ratio between DNA and RNA could be effective in estimating the number of viable parasites in the sample.

In our study, we developed, for the first time, a simple and fast methodology for the extraction of RNA from açaí pulp, together with one-step RT-qPCR assays, targeting the transcript corresponding to the satDNA region of the *T. cruzi* genome, as well as an internal exogenous amplification control (RNA-IAC), which allows for the validation of true negative samples, monitoring the efficiency of RNA extraction and the absence of inhibitors in the RT-qPCR assays. The RNA-IAC is a synthetic RNA that corresponds to the transcript of the firefly (Coleoptera) luciferase gene [35] that has no homology with plants, triatomine insects, or *T. cruzi*.

The one-step RT-qPCR assay was used for the detection and quantification of viable *T. cruzi* in açaí-based samples as a fast, sensitive, and reproducible methodology. For this, one major step of this standardization includes the mixture of 6M guanidine-HCl/0.2M EDTA (pH 8.0) solution in a 1:1 ratio with açaí pulp samples. Guanidine-HCl is a chaotropic salt that is capable of breaking cells and inhibiting nucleases, facilitating the preservation of genetic material in samples, as already described in studies of molecular diagnosis for CD (DNA) from blood [36,37,38,39] and açai [19]. In RNA extraction, the use of this methodology made it possible to overcome the complexity of the açaí pulp, which is composed of several inhibitors that are capable of reducing the efficiency of the RT-qPCR assay [9,40]. Thus, an extra centrifugation step was included before RNA extraction to separate the supernatant from most of the potential inhibitors without losing sensitivity in the molecular diagnostic. At the end of RNA extraction, a DNase I treatment was also included to eliminate contaminating DNA and ensure the specificity of the molecular detection of *T. cruzi* RNA only. In parallel, no-RT controls were used in each one-step RT-qPCR assay.

To facilitate and the methodology and make it fast and reproducible, we developed a one-step RT-qPCR assay using TaqMan systems. The choice of the molecular target for the detection and quantification of viable *T. cruzi*, the transcript corresponding to the nuclear satellite DNA of *T. cruzi* (satDNA), was based on the large number of studies that used this target for the detection of the parasite’s DNA [19,39,41,42] in qPCR assays due to the high sensitivity and specificity. Therefore, we investigated the ability of the cruzi1 and cruzi2 primers and the cruzi3 probe to detect different *T. cruzi* strains in the one-step RT-qPCR. The inclusivity assay detected three *T. cruzi* DTUs from 10^4^ to 0.1 par. Eq./mL in açai pulp for TcI and TcIV, while TcIII was detected up to 10 par. Eq./mL. Considering the particular polycistronic transcription mechanism found in the tripanosomatids, this lower sensitivity to TcIII could correspond to a lower amount of satDNA transcripts in this strain due to a modulation of gene transcription or a lower number of satDNA copies in the parasite genome [43,44,45]. In addition, it is important to point out that the RT-qPCR controls tested without the inclusion of a reverse transcriptase enzyme in the assay (no-RT control) did not result in qPCR amplification, confirming the absence of contaminant DNA in our samples due to the treatment of the RNA with DNaseI.

In another step of the study, precision parameters were evaluated using the Ct values detected in the repeatability assay. In the three low concentrations tested, high repeatability was observed through the small coefficient of variations observed, which were below 2% except for the test with a concentration of 0.5 Eq. Par/mL that had a CV value equal to 2.01%. The validation in the one-step RT-qPCR assay with samples of açaí pulp from the municipality of Coari (Amazonas State, Brazil) showed that eight out of the nine samples evaluated were positive for T. cruzi DNA and RNA. From the RNA/DNA ratio analysis [46], we evidenced the viability of the parasites in the samples, which ranged from 5.80 to 100%. This means that, in the original samples collected in 2018 [19], there was a fraction of viable parasites in the açai pulps, which could be a risk for oral transmission in that occasion. Even a few years later, it was possible to detect RNA in the samples that were collected in 2018, probably due to the addition of a preservation buffer (guanidine-EDTA) associated with storage at a temperature of −80 °C. However, it is important to mention that one of the nine samples that were positive for *T. cruzi* DNA in 2018 [19] showed no amplification for DNA and RNA in the present study, probably due to the presence of PCR inhibitors or the degradation of the nucleic acids, even in the same storage conditions.

Finally, according to the 2018 Technical Note by the Brazilian Agricultural Research Corporation and studies to control pathogens such as *T. cruzi* [16], thermal shock known as bleaching must be performed with the immersion of the fruit in water at a temperature between 80 °C and 90 °C for 10 s and then in water at room temperature until the açai berries cool, thus inactivating the protozoa and other pathogenic microorganisms that may be present in the fruits [16]. For this reason, in this work, we performed the detection of the parasite’s genetic material after bleaching the contaminated açai berries. As expected, it was possible to detect *T. cruzi* DNA during the entire experimental analysis (3 days). However, *T. cruzi* RNA was not detected even just after bleaching (time zero) or at any time during the analysis. Both exogenous internal amplification controls (exo-IPC for DNA and RNA-IAC for RNA) resulted in expected Ct values, showing no qPCR or RT-qPCR inhibition or any issues during the DNA or RNA extraction and validating the results. Accordingly, this result reinforces the importance of the *T. cruzi* RNA analysis to detect viable parasites in açaí samples and the implementation of good manufacturing practices, such as bleaching, which was effective in eliminating viable parasites from açai berries.

Several studies suggest the potential of RNA as a viability marker when evaluating microorganisms in different samples, such as *Plasmodium falciparum*, *Leishmania* sp., *Toxoplasma gondii*, and *Mycobacterium tuberculosis* [28,30,47]. Also, it was possible to detect *T. cruzi* RNA in food samples using RT-qPCR [21,32]. Herein, we used the same molecular target to quantify the parasite’s DNA or RNA, which allowed us to make a more accurate comparison between the quantification of total and viable parasites. By using this target, we obtained a wide dynamic range in the one-step RT-qPCR, with an elevated coefficient of determination and a higher PCR efficiency. Most importantly, this assay showed a high sensitivity as it was capable of detecting up to 0.1 par. Eq./mL. Compared to the RNA detection methodology for *T. cruzi* in the açaí samples previously reported [21,32], our methodology presents significant advantages, such as (i) a 10 times higher sensitivity for *T. cruzi* from DTUs TcI and TcIV; (ii) the preservation of açaí samples in guanidine–EDTA buffer, which preserves nucleic acids and allows for sample transportation at room temperature and storage at 4 °C [36]; (iii) DNA and RNA extraction using commercial silica column kits in a faster and more reproducible manner; (iv) the use of exogenous internal controls, differentiating true negative results from false negatives and controlling the quality of DNA extraction and RT-qPCR; (v) faster (one-step) RT-qPCR assays using TaqMan probes, which are considered the gold standard for molecular diagnosis by qPCR; and (vi) validation using field-collected samples from a municipality with a history of Chagas disease outbreaks through oral transmission.

Together, the results from the present study support the potential of molecular diagnosis tools to be used for the epidemiological vigilance of Chagas disease outbreaks through oral transmission by the consumption of açai pulp or juice, and they raise a new methodological approach to supervise the handling of the fruit during the production chain, which corresponds to an urgent need for the industry of açai. Even with this substantial advancement, the authors acknowledge that a new study still needs to be conducted with a larger number of samples of açaí and other foods involved in oral outbreaks of Chagas disease to further validate the methodology and compare it with other molecular methodologies.

## 4. Materials and Methods

### 4.1. Açaí Samples

The açaí fruit used in the analytical validation and subjected to bleaching was purchased at the Ver-O-Peso market in the city of Belém, located in the state of Pará, Brazil. The fruit was transported and stored at −20 °C until use.

### 4.2. Trypanosoma cruzi Cultivation

The *T. cruzi* epimastigotes strains/clones Dm28c (TcI), INPA 3663 (TcIII), and INPA 4167 (TcIV) were obtained from the Oswaldo Cruz Foundation Protozoan Collection (Fiocruz, COLPROT). Epimastigotes were cultured in LIT medium (Liver infusion Tryptose (BD, Franklin Lakes, NJ, USA) supplemented with 10% fetal bovine serum at 28 °C. In the logarithmic phase of growth, the parasites were recovered by centrifugation (3000× *g* for 10 min at 4 °C), washed three times with 0.15 M NaCl and 0.01 M phosphate buffer pH 7.2 (PBS), and resuspended in 500 µL in the same solution. Cell growth was estimated by counting cells in a Neubauer Chamber (parasites/mL) and used in the artificial contamination of açaí pulp samples.

### 4.3. Preparation of Guanidine-EDTA Açaí (GEA) Samples

For the linearity, sensitivity, and exclusivity analyses, *T. cruzi*-negative açaí pulp samples were provided by the National Institute for Quality Control in Health (INCQS/FIOCRUZ), Rio de Janeiro, Brazil. The samples were separated into 5 mL aliquots and mixed with a lysis solution containing 6 M of guanidine-HCL 0.2 N EDTA in a 1:1 volume ratio. The açaí lysate (GEA) was spiked with different concentrations of *T. cruzi* reference strains belonging to the DTUs I, III, and IV and centrifuged at 15,000× *g* for 20 min at 25 °C. GEA supernatants were recovered and stored at −80 °C until DNA and RNA extraction.

### 4.4. DNA and RNA Extraction

DNA was extracted from GEA samples as described in the study by Finamore-Araujo et al., 2021 [19]. Briefly, 300 µL of GEA supernatant was extracted using the high-purity PCR template preparation kit (Roche Life Science, Mannheim, Germany) according to the manufacturer’s instructions, with some modifications such as changing the sample volume from 200 to 300 μL, using a longer time for the proteinase K lysis step (2 h at 56 °C), and including 2 μL of TaqMan Exogenous Internal Positive Control Reagents (Applied Biosystems, Foster City, CA, USA) per sample prior to extraction. Exo-IPC DNA is a synthetic molecule that does not show homology with any DNA sequence available in public databases. The DNA samples were eluted in 100 μL of elution buffer and stored at −20 °C.

**Figure 6:**
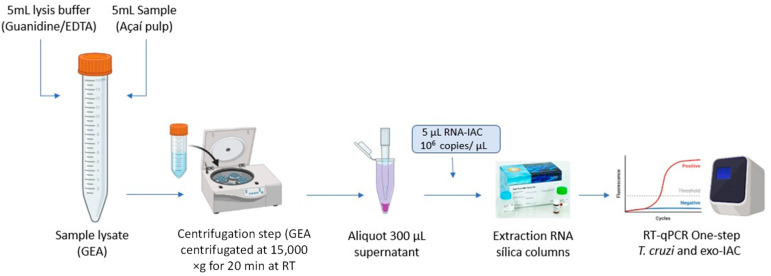
Experimental design of sample preparation of açaí guanidine–EDTA (GEA) and RNA extraction using Exogenous Internal Positive Control (RNA-IAC). Scheme shows RNA extraction from GEA supernatant spiked with exo-IAC synthetic RNA using commercial silica column-based kit followed by one-step RT-qPCR.

As with DNA, RNA was extracted from 300 µL of GEA supernatant using the High Pure RNA Tissue Kit (Roche, Basel, Switzerland) according to the manufacturer’s instructions with some modifications. Five microliters of RNA Luciferase Control (Promega, Madison, WI, USA) at 10^6^ RNA transcripts/µL) was added to the GEA supernatant as an exogenous internal amplification control (RNA-IAC) since the sequence of this RNA molecule has no homology with parasites, triatomines, or plant insects (Figure 6). In addition, the volume of lysis/binding buffer was reduced by half (200 µL). In the RNA elution step, a 50 µL volume of elution buffer was used to elute the total RNA, and the samples were stored at −80 °C. For each RNA extraction run with up to 11 samples, a negative control was included. A sample was considered valid when the RNA-IAC target was amplified with the expected Ct (Ct) values for the same extraction batch. Finally, the purity and concentration of the samples (DNA and RNA) were estimated using a NanoDrop 2000c spectrophotometer (Thermo Fisher Scientific, Waltham, MA, USA) at 260/280 and 260/230 nm.

### 4.5. One Step RT-qPCR for T. cruzi Detection and Quantification

For DNA detection and quantification, real-time multiplex PCR assays were performed as described in the study by Finamore-Araujo et al., 2021 [19]. Briefly, the reactions were performed in a final volume of 20 μL containing 5 μL of DNA as template, 10 µL of 2× FastStart TaqMan^®^ Probe Master Mix (Roche Life Science, Mannheim, Germany), 750 nM of cruzi 1 and cruzi 2 primers, 250 nM of cruzi 3 probe targeting *T. cruzi* nuclear satellite DNA (satDNA), and 1 μL of 10× exo-IPC Mix from the commercial TaqMan Exogenous Internal Positive Control Reagents kit (Applied Biosystems, Foster City, CA, USA), which contains a primer and probe set targeting synthetic DNA exo-IPC. The cycling conditions were as follows: 50 °C for 2 min, 95 °C for 10 min, followed by 45 cycles at 95 °C for 15 s and 58 °C for 1 min.

For one-step RT-qPCR, 2 different targets, *T. cruzi* nuclear satellite transcript (satDNA) and RNA-IAC (as exogenous internal positive control), were evaluated in parallel in singleplex. Reactions were performed in duplicate in a final volume of 10 μL containing 2 μL of RNA extracted with the High Pure RNA Tissue Kit; 5 μL of 2× RT-PCR Buffer and 0.4 μL of 25× RT-PCR Enzyme Mix, both from the AgPath-ID One-Step RT-PCR Reagents kit (Applied Biosystems); 750 nM of cruzi 1 and cruzi 2 primers; and 50 nM of cruzi 3 probe (FAM/NFQ-MGB). For the target in the exogenous internal positive control, we used 700 nM of exo-IAC forward and reverse primers and 200 nM of exo-IAC probe (VIC/NFQ-MGB). One-step RT-qPCR was performed on the QuantStudio Real-Time PCR systems equipment (Applied Biosystems), and the following cycling conditions were used: 45 °C for 15 min (reverse transcription), 95 °C for 5 min (denaturation), followed by 45 cycles at 95 °C for 15 s and 58 °C for 30 s for annealing/extension. The sequences of all sets of primers and probes are described in Table 4.

### 4.6. Inclusivity Assay

To assess the ability of the technique to detect *T. cruzi* RNA in GEA samples and for the development and standardization of RT-qPCR, RNA from a representative panel of strains/clones belonging to three different *T. cruzi* DTUs was tested: Dm28c (TcI), INPA 3663 (TcIII), and INPA 4167 (TcIV). In the assay, samples were tested in duplicate at a concentration ranging from 0.01 to 10^4^ parasite equivalents/mL in singleplex one-step RT-qPCRs targeting *T. cruzi* and RNA-IAC.

### 4.7. Sensitivity Assay

In order to evaluate the analytical sensitivity for RNA detection, the GEA samples were artificially infected with *T. cruzi* Dm28c (Tc I) and serially diluted from 5 to 0.1 parasite equivalents/mL prior to GEA supernatant isolation and RNA extraction. In addition, 300 μL aliquots of GEA supernatant were spiked with RNA-IAC before RNA extraction, and amplifications of *T. cruzi* and exo-IAC targets were monitored using Ct values in RT-qPCR assays.

### 4.8. Standard Curves Preparation

Parasite load quantification assays were performed based on absolute quantification using a standard curve for DNA and RNA. To generate the curves, GEA was added to a pool of *T. cruzi* before the extraction steps, serial dilution of DNA or RNA was performed at a 1:10 ratio, and negative GEA RNA or GEA DNA was used as diluent. Concentrations ranged from 10^5^ to 1 equivalent of *T. cruzi*/mL, and each point on the standard curve was tested in duplicate and added to all qPCR or RT-qPCR plates.

### 4.9. Validation with Açaí Pulp Samples from Municipality of Coari (AM)

Previously [20], we validated a multiplex qPCR assay targeting *T. cruzi* satDNA and an exogenous internal positive control (exo-IPC) using 45 samples of açaí pulp collected in the city of Coari (AM), which were analyzed for the presence of *T. cruzi* DNA following a standardized, easily reproducible, and sensitive methodology, which made it possible to detect and quantify *T. cruzi* in 9 samples (20%) through multiplex qPCR. In the present study, these nine positive samples underwent RNA extraction. Next, we used the one-step singleplex RT-qPCR targeting *T. cruzi* RNA and RNA-IAC to evaluate the viability of the parasite through the detection and quantification of RNA. Still, using the ratio between the average parasite load (DNA/RNA), it was possible to evaluate the percentage of viable parasites in the açaí pulp samples using the following formula: viable *T. cruzi* (%) = [Total *T. cruzi* load (DNA quantification) ÷ living *T. cruzi* load (RNA quantification)] × 100.

During the entire time interval, the samples remained properly preserved in 6 M of guanidine–HCl/0.2 M EDTA buffer and stored at −80° C. All samples were tested in duplicate in both detection methods (qPCR and RT-qPCR).

### 4.10. Açaí Fruit Bleaching

Feces of fifth instar nymphs of *Rhodnius prolixus* that were artificially fed on defibrinated rabbit blood was collected from 0 to 72 h after feeding using a tube with the bottom cut over another 1.5 mL tube. Defibrinated rabbit blood was provided by the Instituto de Ciência e Tecnologia em Biomodelos (ICTB, Fiocruz) following the Ethical Principles in Animal Experimentation and approved by the Comissão de Ética no Uso de Animais (CEUA/Fiocruz, under the protocol number LW019/17). Feces from uninfected triatomines was collected in 1.5 mL tubes and spiked with *T. cruzi* clone Dm28c to reach a concentration of 10^3^ parasites/mL. Subsequently, açaí berries, purchased at the Ver-O-Peso market (Belém/PA, Brazil), were contaminated with feces containing *T. cruzi* by pipetting the feces onto the fruit surface. After drying the feces for 30 min at room temperature, the açaí berries were bleached. Bleaching started with the complete immersion of the fruits in water at a temperature of 80 °C for 10 s. Then, we carried out thermal shock by removing the fruit from the water at a temperature of 80 °C and immediately immersing it in water at room temperature. Once this was complete, the açaí pulp was extracted by processing the fruit, which involved breaking it up using a blender. The resulting pulp was then filtered through a sieve in order to separate the larger elements, thus ensuring that the pulp was obtained in its pure form.

The extracted pulp was stored at two different temperatures (4 °C and −20 °C) for 3 days. At 0 (immediately after pulp removal), 1, and 3 days after, the samples were mixed with 6 M of guanidine–HCL/0.2 N EDTA in a 1: 1 ratio (guanidine–HCL EDTA-açai: GEA) and then centrifuged at 15,000× *g* for 20 min at 25 °C for RNA extraction and 10,000× *g* for 10 min at 25 °C for DNA extraction. Then, qPCR (targeting *T. cruzi* DNA) and one-step RT-qPCR (targeting *T. cruzi* RNA) assays were performed as described.

## 5. Conclusions

Based on this study, it was possible to standardize a method of RNA extraction using a simple and reproducible methodology. We used an internal amplification control to differentiate false negative samples from true negative samples and a chaotropic salt capable of rupturing cells and inhibiting nucleases, making it easier to preserve the genetic material in the samples (guanidine–EDTA buffer). In addition, the extra centrifugation steps made it possible to eliminate inhibitors present in the açaí pulp samples that could interfere with the final yield.

One-step RT-qPCR assays using the TaqMan system for the satDNA target proved to be promising for detecting and quantifying the parasite’s RNA, with high linearity (r^2^ = 0.99) and a reaction efficiency of 88.35%. In addition, RT-qPCR was able to detect three different DTUs of *T. cruzi* when the inclusivity assay was carried out and demonstrated considerable sensitivity as it detected up to 0.1 viable parasite equivalents/mL in the açaí samples.

Finally, it was possible to detect the parasite’s genetic material (DNA) immediately after bleaching the infected açaí, but not its viability (RNA). In other words, it was not possible to detect *T. cruzi* RNA at any point in the analysis after bleaching. To validate the one-step RT-qPCR, we used nine açaí samples that were positive for *T. cruzi* DNA collected in a municipality with a history of oral CD outbreaks (Coari-AM). Eight of these samples were positive for *T. cruzi* RNA, validating the methodology for the detection and quantification of viable parasites in the samples collected in the field.

## Figures and Tables

**Table 1:** Analytical sensitivity for the detection of viable *T. cruzi* (RNA) in GEA samples. RNA was extracted from GEA samples spiked with *T. cruzi* (Dm28c) ranging from 5 to 0.1 *T. cruzi* equivalents/mL. In all samples, 5 μL of RNA-IAC was added before RNA extraction. The results are shown as the Ct mean ± SD for *T. cruzi* and RNA-IAC in one-step RT-qPCR assays.

*T. cruzi* (Par. Eq/mL)	*T. cruzi* Ct Mean ± SD	RNA-IAC Ct Mean ± SD
5	28.45 ± 0.38	22.52 ± 0.06
1	32.39 ± 0.13	23.27 ± 0.01
0.5	32.31 ± 0.15	23.29 ± 0.10
0.1	34.27 ± 0.01	23.54 ± 0.28

**Table 2:** An inclusivity assay for the detection of *T. cruzi* from different DTUs. RNA was obtained from a panel of GEA samples spiked with *T. cruzi* from three different DTUs (TcI, TcIII, and TcIV). The RNA concentrations ranged from 10^4^ to 10^−1^ Par. Eq./mL. The results are shown as the Ct mean ± SD obtained from duplicates of each RNA concentration.

Concentration (Par. Eq/mL)	Tc I		Tc III		Tc IV	
	Ct Mean ± SD		Ct Mean ± SD		Ct Mean ± SD	
	*T. cruzi*	RNA-IAC	*T. cruzi*	RNA-IAC	*T. cruzi*	RNA-IAC
10^4^	20.83 ± 0.98	22.98 ± 0.18	23.69 ± 1.74	24.74 ± 0.28	23.43 ± 0.56	23.73 ± 0.50
10^3^	24.58 ± 0.25	22.40 ± 0.20	26.62 ± 0.23	27.14 ± 0.35	27.23 ± 0.49	24.86 ± 0.80
10^2^	26.43 ± 0.47	22.67 ± 0.25	30.90 ± 1.99	25.00 ± 0.70	31.78 ± 0.49	25.45 ± 0.17
10	26.74 ± 0.12	22.55 ± 0.12	34.82 ± 0.84	26.05 ± 0.0	32.84 ± 1.24	25.74 ± 0.98
1	28.49 ± 0.92	23.80 ± 0.34	NA ^1^	25.12 ± 0.44	34.51 ± 1.63	25.55 ± 0.4
0.1	29.36 ± 0.51	23.53 ± 0.67	NA ^1^	26.85 ± 0.05	40.08 ± 4.31	26.89 ± 0.35

^1^ NA: no amplification.

**Table 3:** The precision (repeatability) of the one-step RT-qPCR assay targeting *T. cruzi*. RNAs extracted from GEA spiked with *T. cruzi* were tested in 40 technical replicates by the same operator at concentrations of 2, 1, and 0.5 Par. Eq./mL.

Sample Concentration	2 Par. Eq/mL	1 Par. Eq/mL	0.5 Par. Eq/mL
Positive results	100%	100%	97.50%
Ct mean (±SD)	30.91 (0.54)	32.07 (0.48)	32.47 (0.65)
Coefficient of variation	1.74%	1.49%	2.01%

**Table 4:** Sets of primers and probe sequences for multiplex qPCR and one-step RT-qPCR assay.

Target	Primers/Probes	Sequences	Amplicon size	Reference
*T. cruzi* (FAM/NFQ-MGB)	Cruzi 1 (Forward)	5′-AAT CGG CTG ATC GTT TTC GA-3′	165 bp	Duffy et al., 2013 [41]
	Cruzi 2 (Reverse)	5′-AAT TCC TCC AAG CAG CGG ATA-3′		
	Cruzi 3 (Probe)	5′-CAC ACA CTG GAC ACC AA-3′		
exo-IPC (VIC/TAMRA)	Not Available	Not Available	Not Available	Applied Biosystems (Cat. Number: 4308323), Finamore-Araujo et al., 2021 [19]
RNA-IAC (VIC/NFQ-MGB)	Forward	5′-TAC AAC ACC CCA ACA TCT TCG A-3′	Not Available	Promega (Cat. Number: L4561), Johnson et al., 2005 [35]
	Reverse	5′-GGA AGT TCA CCG GCG TCA T-3′		
	Probe	5′-CGG GCG TGG CAG GTC TTC CC-3′		

## Data Availability

The original contributions presented in this study are included in the article/Supplementary Material; further inquiries can be directed to the corresponding author/s.

## Supplementary Materials

The supporting information can be downloaded at https://www.mdpi.com/article/10.3390/ijms25105531/s1.
