# Supplementary material for: The Development of a One-Step RT-qPCR for the Detection and Quantification of Viable Forms of Trypanosoma cruzi in Açai Samples from Areas at Risk of Chagas Disease through Oral Transmission
Source: Int J Mol Sci. 2024 May 18;25(10):5531. doi: 10.3390/ijms25105531 (PMC11122307; doi:10.3390/ijms25105531)
Supplement: Supplementary file 1 [file ijms-25-05531-s001.zip › ijms-2987685-supplementary.pdf]

Table S1. Ct values for the Comparison of parasite load quantified using *T. cruzi* DNA and RNA in açai pulp samples after bleaching.

Sample	DNA				RNA			
	Ct Mean (Sat-DNA)	*SD*	*Ct Mean (exo-IPC)*	*SD*	*Ct Mean (Sat-DNA)*	*SD*	*Ct Mean (RNA-IAC)*	*SD*
**Day 0 (4°C)**	35.85	0.12	31.72	0.35	NA	NA	27.54	0.67
**Day 1 (4°C)**	40.22	0.23	32.20	0.17	NA	NA	28.21	0.35
**Day 3 (4°C)**	38.23	0.44	31.36	0.26	NA	NA	27.89	0.24
**Day 0 (-20°C)**	33.99	0.28	31.77	0.29	NA	NA	27.63	0.28
**Day 1 (-20°C)**	39.71	0.25	32.79	0.33	NA	NA	28.14	0.51
**Day 3 (-20°C)**	39.11	0.71	31.13	0.06	NA	NA	28.37	0.29

Ct: Threshold Cycle. SD: Standard deviation.
